# Anti-Herpes Simplex Virus and Anti-Inflammatory Activities of the Melittin Peptides Derived from *Apis mellifera* and *Apis florea* Venom

**DOI:** 10.3390/insects15020109

**Published:** 2024-02-04

**Authors:** Pichet Praphawilai, Thida Kaewkod, Sureeporn Suriyaprom, Aussara Panya, Terd Disayathanoowat, Yingmanee Tragoolpua

**Affiliations:** 1 Department of Biology, Faculty of Science, Chiang Mai University, Chiang Mai 50200, Thailand; pichet.p@cmu.ac.th (P.P.); thida.kaewkod@cmu.ac.th (T.K.); sureeporn.suriyaprom@cmu.ac.th (S.S.); aussara.pan@cmu.ac.th (A.P.); terd.dis@cmu.ac.th (T.D.); 2 Office of Research Administration, Chiang Mai University, Chiang Mai 50200, Thailand; 3 Research Center of Deep Technology in Beekeeping and Bee Products for Sustainable Development Goals (SMART BEE SDGs), Faculty of Science, Chiang Mai University, Chiang Mai 50200, Thailand

**Keywords:** anti-herpes simplex virus, anti-inflammation, bee venom, herpes simplex virus, melittin, plaque reduction assay, virucidal assay

## Abstract

**Simple Summary:**

Herpes simplex viruses are classified into herpes simplex virus types 1 (HSV-1) and 2 (HSV-2) and cause infectious diseases ranging from oral and genital herpes to severe conditions like encephalitis. Transmission occurs through contact with infected secretions, leading to primary infections and subsequent latency. The current antiviral drugs, such as acyclovir, inhibit viral replication but do not prevent recurrence and may lead to drug-resistant strains. Bee venom, particularly the melittin from *Apis mellifera*, has been used in folk medicine for its antimicrobial and anti-inflammatory properties. Melittin disrupts lipid membranes, exhibiting antibacterial, antifungal and antiviral activities. In this study, melittin peptides from *A. mellifera* (MEL-AM) and *Apis florea* (MEL-AF) were compared and their inhibitory effects on HSV-1 and HSV-2 using plaque reduction and virucidal assays. Additionally, the anti-inflammatory effects in LPS-stimulated macrophages are explored and melittin emphasized as a potential antiviral and anti-inflammatory agent.

**Abstract:**

Herpes simplex virus (HSV) is known to cause cold sores and various diseases in humans. Importantly, HSV infection can develop latent and recurrent infections, and it is also known to cause inflammation. These infections are difficult to control, and effective treatment of the disease remains a challenge. Thus, the search for new antiviral and anti-inflammatory agents is a necessity. Melittin is a major peptide that is present in the venom of the honeybee. It possesses a number of pharmacological properties. In this study, the effects of the melittin peptides from *A. mellifera* (MEL-AM) and *A. florea* (MEL-AF) against HSV-1 and HSV-2 were evaluated at different stages during the viral multiplication cycle in an attempt to define the mode of antiviral action using plaque reduction and virucidal assays. The results revealed a new finding that melittin at 5 µg/mL demonstrated the highest inhibitory effect on HSV through the direct inactivation of viral particles, and MEL-AF displayed a greater virucidal activity. Moreover, melittin was also observed to interfere with the process of HSV attachment to the host cells. MEL-AM exhibited anti-HSV-1 and anti-HSV-2 effects with EC_50_ values of 4.90 ± 0.15 and 4.39 ± 0.20 µg/mL, while MEL-AF demonstrated EC_50_ values of 4.47 ± 0.21 and 3.95 ± 0.61 µg/mL against HSV-1 and HSV-2, respectively. However, non-cytotoxic concentrations of both types of melittin produced only slight degrees of HSV-1 and HSV-2 inhibition after viral attachment, but melittin at 5 µg/mL was able to reduce the plaque size of HSV-2 when compared to the untreated group. In addition, MEL-AM and MEL-AF also exhibited anti-inflammatory activity via the inhibition of nitric oxide production in LPS-stimulated RAW 264.7 macrophage cells, and they were also found to down-regulate the expressions of the *iNOS*, *COX-2* and *IL-6* genes. The highest inhibition of *IL-6* mRNA expression was found after treatment with 10 µg/mL of MEL-AM and MEL-AF. Therefore, melittin peptides have displayed strong potential to be used as an alternative treatment for HSV infection and inflammatory diseases in the future.

## 1. Introduction

Herpes simplex viruses are enveloped viruses that contain double-stranded DNA genomes. These viruses belong to the family *Herpesviridae*, subfamily *Alphaherpesvirinae*. The important human herpes simplex viruses can be divided into two distinct antigenic types: herpes simplex virus type 1 (HSV-1) and herpes simplex virus type 2 (HSV-2). These viruses are known to be the cause of human herpes, which is associated with a variety of mild to severe diseases such as oral and genital herpes, herpes keratitis, herpes conjunctivitis, herpetic encephalitis, eczema herpeticum and neonatal herpes [1]. The general spread of a herpes infection occurs from direct close contact with infected secretions. HSV-1 is usually transmitted through direct contact with infectious secretions or lesions on the face, lips, mouth cavity and skin area, all of which are associated with orofacial disease. Alternatively, HSV-2 has been traditionally associated with genital diseases through contact with lesions or secretions of infected skin around the genital area as a result of sexual activity [2]. Both types of HSVs can be transmitted by symptomatic and asymptomatic individuals. After primary infection, HSV can avoid the host immune system by entering a non-replicating state referred to as latency infection. The virus can then replicate itself after being reactivated by various forms of environmental stimulation [3,4]. The standard antiviral drugs that are used in the treatment of herpetic infections are based on the use of modified nucleosides or their derivatives, such as acyclovir. The main action of acyclovir involves the inhibition of viral DNA polymerase and viral replication, but these drugs do not prevent the recurrence of infection. Moreover, the long-term usage of antiviral drugs may promote resistant strains of HSV and can also cause certain undesirable side effects in some patients [5]. The lesions associated with herpes infection often result in secondary bacterial infections and extensive inflammation that may require complicated forms of treatment [6]. Normally, inflammation is a primary response to any stimuli such as pathogens, irritants and damage to cells or tissue. Stimulation agents, such as viral infection, are able to trigger macrophage cells to produce proinflammatory mediators. These responses mainly contribute to the elimination of the stimulation agent and initiate the healing process [7]. However, extensive inflammatory responses may lead to a variety of chronic inflammatory diseases. Therefore, studies that focus on the search for novel and effective antiviral and anti-inflammation agents are considered indispensable. It has been widely reported that animal venom is a rich source of antimicrobial substances. The venom of animals is a mixture of compounds that are mostly made up of peptides. These compounds are known to exhibit a broad range of biological activities [8].

Bee venom or apitoxin has long been used in folk medicine for the treatment of many diseases and conditions, such as wounds, inflammation, infections and cancer [9,10]. It consists of a mixture of proteins, peptides and low-molecular-weight components such as phospholipase A_2_ (PLA_2_), hyaluronidase, melittin, apamine, mast cell degranulating peptide (MCD), adolapin, histamine, serotonin and catecholamine [11]. Melittin is a major peptide in honeybee venom. It makes up 40–50% of the dry weight of bee venom. A molecule of melittin consists of 26 amino acids. Predominant peptide is the hydrophobic N-terminal region, while the C-terminal part of the molecule is positively charged. This distribution leads melittin to be an amphiphilic peptide [12,13]. Melittin is bound to a negative charge on the lipid membrane of cells or pathogens and can disturb the stability of phospholipid bilayers by forming pores. It induces the leakage of atomic ions and molecules and leads to cell lysis [14]. The melittin peptide has demonstrated a number of diverse properties such as antibacterial, antifungal and antiviral activities, as well as a range of pharmacological activities, such as anti-inflammatory and anticancer properties [15,16,17]. Many reports have indicated that the melittin delivered by the western honeybee *A. mellifera* (MEL-AM) is able to inhibit a broad range of viruses through various mechanisms. Melittin impeded the multiplication of the Junin virus (JV) and the herpes simplex viruses [18]. Moreover, melittin could inhibit the replication of Human Immunodeficiency Virus type 1 by suppressing viral gene expression [19]. Meanwhile, at the same time, the antiviral properties of a related species of honeybee, the red dwarf honeybee *A*. *florea* (MEL-AF), have not been fully explained or reported. Based on this background, melittin is recognized as a compound with a high potential for use as an antiviral agent. Therefore, we have evaluated, in comparison, melittin from *A. mellifera* and *A. florea* in its inhibitory effect on HSV-1 and HSV-2 through various mechanisms of replication in order to define the mode of action using plaque reduction and virucidal assays. Moreover, the anti-inflammatory effects of melittin in LPS-stimulated RAW 264.7 macrophage cells were also observed.

## 2. Materials and Methods

### 2.1. Synthetic Peptides

The melittin peptides from *Apis melifera*, MEL-AM; GIGAVLKVLTTGLPALISWIKRKRQQG and *Apis florea*, MEL-AF; GIGAILKVLATGLPTLISWIKNKRKQG were synthesized by Synpeptide Co., Ltd., Shanghai, China, and the peptides were dissolved in deionized water. A stock solution of the melittin peptides (10 mg/mL) was aliquoted and stored at −20 °C.

### 2.2. Cell Culture

The Vero cells and RAW 264.7 cells were grown in Dulbecco’s modified Eagle’s medium (DMEM) (Gibco, Grand Island, NY, USA) supplemented with 10% fetal bovine serum (Hyclone, Logan, UT, USA), penicillin (100 units/mL) and streptomycin (100 µg/mL). The cells were incubated at 37 °C in a 5% CO_2_ humidified atmosphere. The RAW 264.7 macrophage cell line originated from mice (*Mus musculus*) and was purchased from ATCC with the accession number ATCC-TIB-71.

### 2.3. Virus Propagation

Herpes simplex virus type 1 strain F (HSV-1F) and herpes simplex virus type 2 strain G (HSV-2G) were propagated on the Vero cells. The viruses were harvested and stored at −80 °C.

### 2.4. Cytotoxicity Assay

The cytotoxicity of melittin was evaluated in the Vero cells and RAW 264.7 cells using an MTT assay. The Vero cells were seeded with a density of 1 × 10^4^ cells per well. Additionally, the RAW 264.7 cells at 1 × 10^5^ cells were seeded in 96-well plates. They were then grown to confluence for 24 h. The cells were then treated with the indicated concentrations of melittin that were prepared in the DMEM without serum in triplicate wells. The treated Vero and RAW 264.7 cells were incubated at 37 °C in a 5% CO_2_ humidified atmosphere for 72 and 24 h, respectively. The percentage of cell viability was then calculated by comparing the resulting values with those of an untreated group without melittin.

### 2.5. Plaque Titration Assay

The HSV viral titers were quantified using a plaque titration assay. The HSV suspensions were diluted in DMEM (10-fold dilution). Each dilution of the virus was then inoculated into confluent monolayer Vero cells and incubated at room temperature for 1 h. The infected cells were covered with overlayer media containing 1.5% carboxymethyl cellulose in a growth medium at the ratio of 1:3 and further incubated at 37 °C in a 5% CO_2_ humidified atmosphere for 48–72 h. The plaques were visualized using crystal violet staining, and the values were calculated as plaque-forming units per milliliter (PFU/mL).

### 2.6. Mode of the Inhibitory Effect of Melittin on HSV

The inhibitory effects of melittin on HSV-1 and HSV-2 were evaluated during the viral multiplicity cycle.

#### 2.6.1. Pretreatment of Cells with Melittin Prior to Virus Infection

The Vero cells were grown to confluence on the monolayer in 24-well plates for 24 h. The Vero cells were then treated with a serial two-fold dilution of melittin (1.25–5 µg/mL) at 37 °C for 1 h. Next, the HSVs at a concentration of 300–400 pfu/mL were used since this concentration was suitable for plaque-counting in 24-well plates. The HSVs were adsorbed onto the treated cells for 1 h at room temperature. The infected cells were covered with overlay media and further incubated at 37 °C in a 5% CO_2_ humidified atmosphere for 48–72 h. The percentage of plaque formation was calculated by comparing the resulting values with untreated cells without melittin.

#### 2.6.2. Treatment of Melittin during HSV Attachment to the Cells

The Vero cells were grown to confluence on the cell monolayer in 24-well plates for 24 h. Serial two-fold dilution of melittin at various concentrations of 1.25–5 µg/mL and the HSVs (300–400 PFU/mL) were added simultaneously to the Vero cells, and they were further incubated at room temperature for 1 h. The infected cells were covered with the overlayer media and incubated at 37 °C in a 5% CO_2_ humidified atmosphere for 72 h. The percentage of plaque formation was calculated by comparing the resulting values with the untreated virus without melittin.

#### 2.6.3. Treatment of Melittin after HSV Attachment to the Cells

The Vero cells were grown to confluence on the cell monolayer in 24-well plates for 24 h. The HSVs (300–400 PFU/mL) were added to the Vero cells, and they were incubated at room temperature for 1 h. The infected cells were treated with melittin at various concentrations of 1.25–5 µg/mL. The treated cells were then covered with the overlayer media and incubated at 37 °C in a 5% CO_2_ humidified atmosphere for 48–72 h. The percentage of plaque formation was calculated by comparing the resulting values with the untreated virus without melittin. In addition, acyclovir was used as the positive control.

#### 2.6.4. Virucidal Effect of Melittin Prior to Virus Infection

The viruses suspended in the indicated titer were co-incubated with equal volumes of melittin (1:1) at 4 °C for 30 min. The treated viruses were diluted using DMEM to eliminate the effects of the remaining melittin on subsequent binding events. The treated viruses were then inoculated into the Vero cells and incubated at room temperature for 1 h. The infectivity of the HSVs was then determined using the plaque titration assay.

### 2.7. The Inflammatory Effect of Melittin on the RAW 264.7 Macrophage Cell Model

The anti-inflammatory effects of melittin were measured according to a nitric oxide reduction assay and determination of the inflammatory gene expression.

#### 2.7.1. Nitric Oxide Reduction Assay

The RAW 264.7 cells were grown to confluence on the cell monolayer in 96-well plates for 24 h. The cells were stimulated with lipopolysaccharide (LPS) (1 µg/mL) for 30 min. The stimulated cells were treated with melittin at concentrations of 2.5–20 µg/mL. The treated cells were then incubated at 37 °C in a 5% CO_2_ humidified atmosphere for 24 h. The supernatant obtained from the treated cells were mixed with Griess reagent followed by further incubation for 10 min at room temperature. The optical density at 550 nm was measured using a microplate reader. The nitrite concentration was calculated using a dilution of sodium nitrite as a standard.

#### 2.7.2. RNA Extraction and Real-Time Quantitative Reverse Transcription Polymerase Chain Reaction (qRT-PCR)

The RAW 264.7 cells were grown to confluence on the cell monolayer in 24-well plates for 24 h and were stimulated using LPS (1 µg/mL) for 30 min. The stimulated cells were treated with melittin at 5 and 10 µg/mL for 3 h. The total RNA from the cells was isolated from the RAW 264.7 macrophages according to the manufacturer’s instructions using NucleoSpin^®^ technology (MACHEREY-NAGEL, Düren, Nordrhein-Westfalen, Germany). The extracted RNA was quantified using a NanoDrop spectrophotometer (Thermo Scientific, Waltham, MA, USA), and then the RNA (1000 ng) was converted into complementary stranded DNA (cDNA) using the reverse transcriptase enzyme ReverTra Ace^TM^ (TOYOBO, Osaka, Japan). Subsequently, the cDNA was amplified via PCR containing 10 μL of SYBR Green PCR mix, 0.8 μL of each primer-specific primer for the inflammatory genes, including *iNOS*, forward 5′-TTCCAGAATCCCTGGACAAGC-3′ and reverse 5′-TGGTCAAACTCTTGGGGTTCG-3′; *COX*-2, forward 5′-AGAAGGAAATGGCTGCAGAA -3′ and reverse 5-GCT CGGCTTCCAGTATTGAG-3′ and *IL-6*, forward 5′-GCTGGAGTCACAGAAGGAGTG-3′ and reverse 5′-GCATAACGCACTAGG TTTGCC-3′ [7,20,21] and 1 μL of the cDNA template to a final reaction volume of 20 μL (SensiFAST™, SYBR® No-ROX Kit (BIOLINE, London, UK). The real-time PCR cycling conditions were applied as follows: 30 cycles of denaturation at 94 °C for 1 min, annealing at 60 °C for 1 min and extension at 72 °C for 1 min. The β-actin forward 5′-TGCTGTCCCTGTATGCCT CTG -3′ and reverse 5′-CTGTAGCCACGCTCGGTCA-3′ primers were used as the internal control [7]. The relative gene expression was calculated using the comparative Ct method (2^−∆∆Ct^) as previously described [22].

### 2.8. Statistical Analysis

The data were analyzed using statistical software applying one-way analysis of variance (ANOVA), followed by multiple comparisons using Duncan’s tests in the SPSS program (version 24.0) with a significant difference established at *p* ≤ 0.05.

## 3. Results

### 3.1. In Vitro Cytotoxicity of Melittin

**Figure 1:**
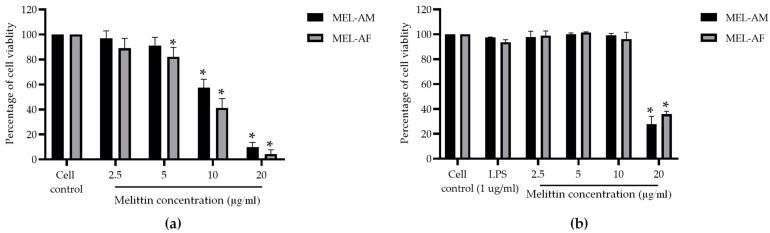
Effect of melittin on cell viability. The percentage of cell viability was measured using MTT assay. The percentage of Vero cell viability was measured after treatment with melittin at various concentrations for 72 h (**a**), as was the effect of melittin on RAW 264.7 cell viability after treatment for 24 h (**b**). Data are presented as the mean percentage of cell viability ± SD from three independent experiments. * Values were significantly different from the untreated group (*p* < 0.05).

The cytotoxicity of melittin at each concentration was tested on the Vero and RAW 264.7 cells. The percentage of cell viability was evaluated using the MTT method. Melittin displayed a cytotoxic effect on the Vero cells in a dose-dependent manner. After 72 h of treatment, MEL-AM and MEL-AF at concentrations above 5 µg/mL significantly reduced the cell viability and induced cell rounding and monolayer detachment. The cytotoxic concentration at 50% (CC_50_) of MEL-AM was 12.05 ± 0.92 µg/mL, while MEL-AF exhibited slightly more toxicity with a CC_50_ value of 10.50 ± 0.91 µg/mL (Figure 1a). Meanwhile, in the RAW 264.7 cells, melittin did not display a cytotoxic effect at concentrations below 10 µg/mL after 24 h of treatment. MEL-AM and MEL-AF exhibited CC_50_ values of 16.69 ± 0.85 and 17.70 ± 0.66 µg/mL, respectively (Figure 1b).

### 3.2. Mode of the Inhibitory Effect of Melittin on HSVs

#### 3.2.1. Pretreatment of Cells with Melittin before HSV Attachment to Cells

The protective effect of melittin against HSV infection was evaluated using melittin at concentrations of 1.25–5 µg/mL on the Vero cell monolayer at 37 °C for 1 h. The results showed that the number of plaques in both the MEL-AM- and MEL-AF-treated groups was not significantly reduced when compared with that in the virally infected cell control group. This result indicated that melittin was unable to prevent the infection of both types of HSVs at the initial stage of viral infection.

#### 3.2.2. Treatment with Melittin during HSV Attachment to Cells

**Figure 2:**
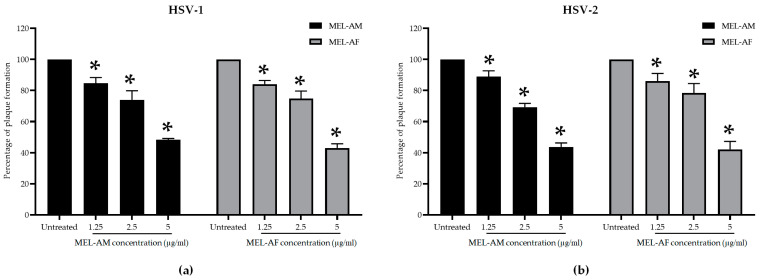
Effect of melittin on HSVs during viral infection. Cells were infected with HSVs and treated with non-cytotoxic concentrations of melittin simultaneously. Relative amounts of plaque formation of HSV-1 (**a**) and HSV-2 (**b**) in the treated groups were compared to those of the untreated group. Data are presented as mean values of relative plaque formation (%) ± SD from three independent experiments. * Values were significantly different from the untreated group (*p* < 0.05).

The interfering capacity of melittin during HSV attachment to the Vero cells was determined by simultaneously adding melittin and the HSVs to the cells. The results showed that both MEL-AM and MEL-AF at the highest non-cytotoxic concentration of 5 µg/mL could inhibit the plaque formation of HSV-1 by more than 50% compared with the untreated control, by 51.76 ± 0.88% and 56.98 ± 2.67%, respectively, and inhibited plaque formation in dose-dependent manner, with EC_50_ values at 4.90 ± 0.15 and 4.47 ± 0.21 µg/mL, respectively (Figure 2a). Moreover, MEL-AM and MEL-AF at 5 µg/mL had anti-HSV-2 activity since the plaque formation was inhibited by 56.32 ± 2.52% and 57.83 ± 5.03%, respectively. The EC_50_ values of MEL-AM and MEL-AF against HSV-2 were 4.39 ± 0.20 and 3.95 ± 0.61 µg/mL, respectively (Figure 2b).

#### 3.2.3. Treatment with Melittin after HSV Attachment to Cells

**Figure 3:**
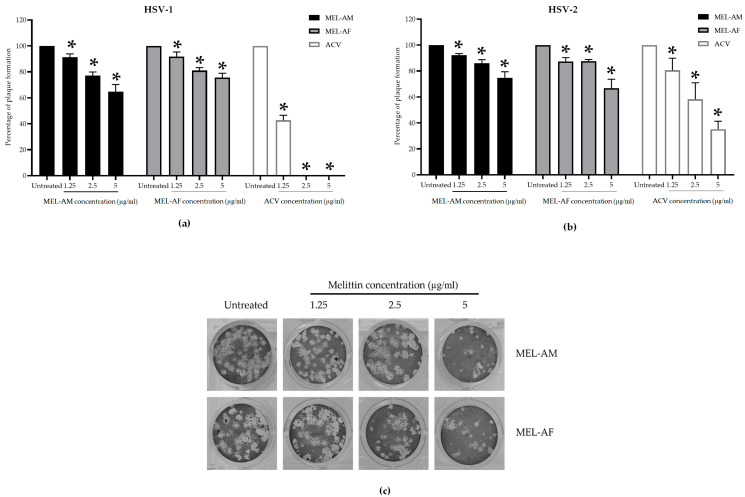
Effect of melittin on HSVs after viral infection. Cells were infected with HSVs followed by being treated with non-cytotoxic concentrations of melittin. The relative amounts of plaque formation of HSV-1 (**a**) and HSV-2 (**b**) in the treated groups were compared with those of the untreated group and ACV positive drug control. The plaque size of HSV-2 was reduced after treatment with melittin at 5 µg/mL (**c**). Data are presented as mean values of relative plaque formation (%) ± SD from three independent experiments. * Values were significantly different from the untreated group (*p* < 0.05).

The efficacy of melittin against HSVs was also determined after viral attachment to the cells. Non-cytotoxic concentrations of melittin at 1.25, 2.5 and 5 µg/mL were added to the HSV-infected cells. The results revealed that the inhibitory effect of melittin on both types of HSVs was less than 50%. The percentage of inhibition of HSV-1 by MEL-AM and MEL-AF was 35.33 ± 5.58% and 24.50 ± 3.44%, respectively (Figure 3a). Likewise, against HSV-2, MEL-AM and MEL-AF exhibited inhibition of 25.41 ± 4.87% and 33.25 ± 6.88%, respectively (Figure 3b). In addition, acyclovir, ACV, was used as a positive drug control and exhibited a higher potential in the inhibition of HSV-1 and HSV-2 with EC_50_ values of 2.05 ± 0.24 and 3.48 ± 0.69 µg/mL. The HSV-1 plaque inhibition was 57.30 ± 3.81, 100.00 ± 0.00 and 100 ± 0.00% when the infected cells were treated with 1.25, 2.5 and 5.0 µg/mL of ACV, respectively (Figure 3a). The HSV-2 plaque inhibition was 19.39 ± 9.22, 41.81 ± 12.73 and 64.83 ± 6.08% when the infected cells were treated with 1.25, 2.5 and 5.0 µg/mL of ACV, respectively (Figure 3b). Although melittin had a low capacity to reduce the number of plaques formed, melittin at 5 µg/mL was able to reduce the plaque size of HSV-2 when compared with the untreated group (Figure 3c).

#### 3.2.4. Virucidal Effect of Melittin Prior to Virus Infection

**Figure 4:**
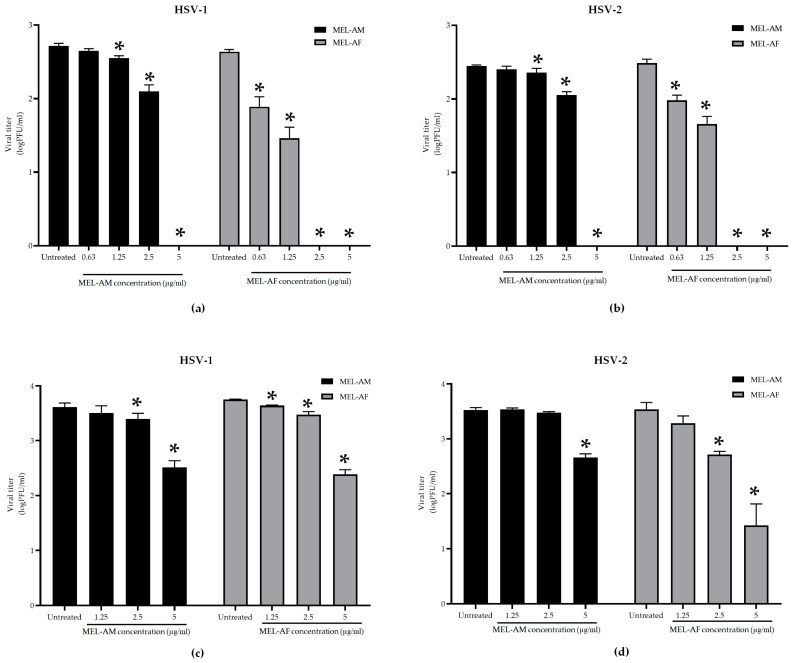
Virucidal effects of melittin on infectivity of HSV-1 and HSV-2. The viral suspension was incubated with melittin at indicated concentrations at 4 °C for 30 min. The infectivity of HSVs was measured using plaque titration assay. HSV-1 and HSV-2 suspensions of 300 to 400 PFU/mL (**a**,**b**) and 3 × 10^3^ to 4 × 10^3^ PFU/mL (**c**,**d**) were treated with non-cytotoxic concentrations of melittin. The titers of HSV-1 and HSV-2 in the treated group were compared to those in the untreated group. Data are presented as mean values of vital titer ± SD from three independent experiments. * Values were significantly different from the untreated group (*p* < 0.05).

The virucidal effect of melittin was achieved using the treatment of the HSVs with melittin at 4 °C for 30 min. The results demonstrated that melittin could inhibit the infectivity of HSV-1 and HSV-2 in a dose-dependent manner. At a low titer of virus (300 to 400 PFU/mL), the plaque formation of the HSVs was inhibited by 100% when treated with MEL-AM at concentrations of 5 μg/mL, while its 50% effective concentrations (EC_50_) were observed to be 1.74 ± 0.02 µg/mL and 2.23 ± 0.12 µg/mL for HSV-1 and HSV-2, respectively. Whereas, MEL-AF showed a higher virucidal effect on HSVs with 100% inhibition at 2.5–5 μg/mL and also showed EC_50_ values of 0.29 ± 0.06 and 0.46 ± 0.07 µg/mL against HSV-1 and HSV-2, respectively (Figure 4a,b). When the titer of the virus was increased (3 × 10^3^ to 4 × 10^3^ PFU/mL), the viral inactivation was slightly reduced after co-incubation with the melittins. The infectivity of HSV-1 and HSV-2 was inhibited by 92.05 ± 1.02% and 86.24 ± 2.02% when treated with MEL-AM at a concentration of 5 μg/mL. This inhibition also presented in a dose-dependent manner with 50% effective concentrations (EC_50_) of 2.85 ± 0.29 and 3.64 ± 0.08 µg/mL against HSV-1 and HSV-2. Meanwhile, MEL-AF had more virucidal effects on both types of HSVs. The highest inhibition of HSV-1 and HSV-2 was observed at 5 μg/mL, 95.63 ± 0.79% and 98.90 ± 0.90%, respectively. The EC_50_ values of MEL-AF against HSV-1 and HSV-2 were 2.67 ± 0.14 and 1.01 ± 0.45 µg/mL. Thus, melittin was found to be able to inactivate particles of HSV-1 and HSV-2 when compared to the untreated group (Figure 4c,d).

### 3.3. Anti-Inflammatory Effect of Melittin on the RAW 264.7 Macrophage Cell Line

#### 3.3.1. Inhibitory Effect of Melittin on Nitric Oxide (NO) Production

**Figure 5:**
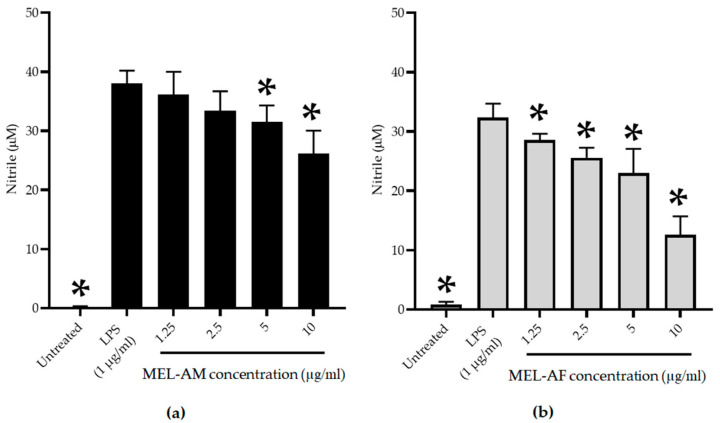
Inhibitory effect of MEL-AM (**a**) and MEL-AF (**b**) at indicated concentrations (1.25–10 µg/mL) on nitric oxide (NO) production in LPS-stimulated RAW 264.7 cells. The cells with or without LPS stimulation were used as the positive control and negative control, respectively. Data are presented as mean ± SD values from three independent experiments. * Values were significantly different from the untreated group (*p* < 0.05).

The RAW 264.7 macrophage cell line was pre-stimulated using LPS (1 µg/mL) for 30 min and the inflammatory effect was determined. The stimulated cells were treated with non-cytotoxic concentrations of melittin (2.5–10 µg/mL) at 37 °C in a 5% CO_2_ incubator for 24 h, and the nitrite was determined as an indirect measurement of the NO production. Melittin displayed an inhibitory effect in a dose-dependent manner. The highest degree of inhibition of inflammation with MEL-AM at 10 µg/mL determined by the NO production was observed at 31.65 ± 6.52% when compared with the up-regulation of nitrite production in the positive control of the LPS-treated cells. Whereas, MEL-AF showed a higher inhibitory effect at 61.84 ± 7.11% and had a 50% inhibitory concentration (IC_50_) of 8.74 ± 1.12 µg/mL (Figure 5).

#### 3.3.2. Inhibitory Effect of Melittin on Pro-Inflammatory Mediator Genes

**Figure 6:**
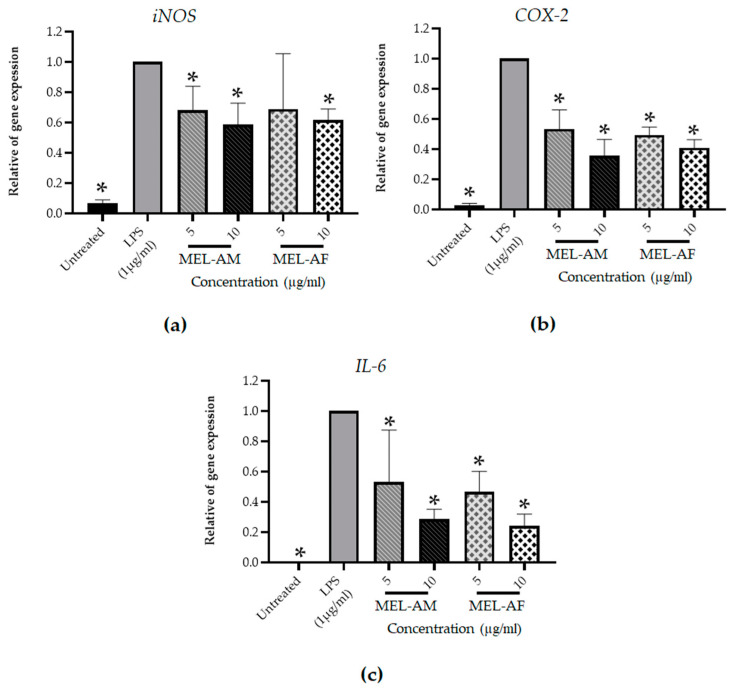
Effect of melittin on the level of pro-inflammatory gene expression after cells were stimulated with LPS (1 µg/mL) and treated with non-cytotoxic concentrations of melittin for 3 h. Expressions of the inflammatory gene were measured using reverse transcription PCR. The relative mRNA expressions of inducible nitric oxide synthase (*iNOS*) (**a**) cyclooxygenase-2 (*COX-2*) (**b**) and interleukin 6 (*IL-6*) (**c**) were normalized to the *β-actin* internal control. The results are representative of those obtained from three independent experiments. * Values were significantly different from the untreated group (*p* < 0.05).

The effect of melittin on the expression of pro-inflammatory mediator genes was determined. Total RNA was isolated from the RAW 264.7 macrophage cells after 3 h of treatment, followed by real-time quantitative reverse transcription PCR (RT-PCR) with the specific primers for the *iNos*, *COX-2* and *IL-6* genes. The real-time quantitative RT-PCR data revealed that the mRNA expression of the *iNos*, *COX-2* and *IL-6* genes increased after 3 h of LPS stimulation of the RAW 264.7 cells compared with the untreated cells (negative control), whereas treatment with 5 and 10 µg/mL of MEL-AM and MEL-AF exhibited down-regulation of the *iNos*, *COX-2* and *IL-6* expression in a dose-dependent manner when compared with the LPS-treated cells (positive control). After treatment of the RAW 264.7 macrophage cells with MEL-AM and MEL-AF at 10 µg/mL, the relative mRNA expression of *iNos* was 0.59 ± 0.14 and 0.62 ± 0.07, respectively. Moreover, the relative mRNA expression of *COX-2* was 0.36 ± 0.11 and 0.41 ± 0.05 after treatment with 10 µg/mL of MEL-AM and MEL-AF, respectively. In addition, the highest inhibition, of *IL-6* mRNA expression, was observed from the relative *IL-6* mRNA expression, 0.29 ± 0.06 and 0.24 ± 0.08 after treatment with 10 µg/mL of MEL-AM and MEL-AF, respectively (Figure 6).

## 4. Discussion

Bee venom has been used as an apitherapy for the treatment of many diseases and conditions such as inflammation, microbial infections and cancer. Bee venom is produced in the venom glands of bees. It is secreted by way of the branched acid gland and the alkaline Dufour’s gland. Thus, this venom consists of a complex mixture of compounds that includes proteins, peptides, amino acids, phospholipids, sugars, biogenic amines, volatile compounds, pheromones and a high quantity of water (>80%) [17,23]. Melittin is the major and the most toxic component of bee venom, constituting 50–60% of the whole venom. The peptide is a α-helical cationic peptide that is composed of 26 amino acid residues. Residues 1–20 of melittin are mainly hydrophobic, while residues 21–26 are more hydrophilic. This has led to the existing amphipathic properties of the peptide. This peptide causes most of the pain associated with bee stings and is known as a lytic peptide. It acts against a wide range of eukaryotic cells by binding with lipid membranes and rapidly leading to membrane disruption and hemolysis. Melittin at high concentrations demonstrated cytotoxicity against both normal and tumor cells [14]. In this study, the cytotoxic effects of melittin from different sources were tested on Vero cells using an MTT assay, and the results showed that both MEL-AM and MEL-AF at concentrations above 5 µg/mL significantly reduced the cell viability and induced changes in the cell morphology. However, the toxicity of melittin was not found in the RAW 264.7 cells in the range of 2.5–10 µg/mL due to the ratio of the surface membrane of the tested cells and the time of incubation. Likewise, several studies have reported that melittin displayed a cytotoxic effect in both dose- and time-dependent manners on different cells and lipid bilayers [24,25,26].

The antiviral activities of melittin against the HSVs at different stages during the viral multiplicity cycle were evaluated in order to clarify the mode of HSV inhibition by the melittin peptides. Pretreatment of the Vero cells with a non-cytotoxic concentration of MEL-AM and MEL-AF prior to infection with the HSVs did not reveal any differences in the amount of plaque formation in the comparison made between the melittin-treated groups and the untreated group. Thus, melittin was found to be unable to, or had a low capacity to, protect the cells from HSV infection. However, both MEL-AM and MEL-AF displayed an excellent inhibitory effect against both types of HSVs through the inactivation of the viral particles. At a low titer of the virus, the plaque formation of the HSVs was inhibited by 100% when treated with melittin at a concentration of 5 μg/mL MEL-AM, whereas MEL-AF showed a higher virucidal effect, with 100% inhibition at 2.5 μg/mL. The infectivity of HSV-1 and HSV-2 was reduced by melittin in a dose-dependent manner. The 50% effective doses (ED_50_) of MEL-AM against HSV-1 and HSV-2 were 1.74 ± 0.02 µg/mL and 2.23 ± 0.12 µg/mL, respectively. A lower ED_50_ was observed in the treatment with MEL-AF, with values of 0.29 ± 0.06 µg/mL for HSV-1 and 0.46 ± 0.07 µg/mL for HSV-2.

These findings indicated that a minor difference in the amino acid sequence in the melittin peptide might affect the stability or interaction of the peptide with viral particles. However, at a higher titer of the virus, the activity of melittin was slightly reduced. The inhibitory effects on HSV were in the range of 80–90% in the treatment of MEL-AM and MEL-AF. These observations indicated that the inhibitory effects of the melittins were dependent upon the initial amount of virus, and our findings suggested that MEL-AF displayed a greater virucidal activity. This effect might be explained by the pore formation of melittin on the lipid bilayer envelope of the HSVs. Melittin was inserted into the lipid membrane, and the self-assembly of the melittin peptide formed a tetramer molecule with a hydrophobic region inside the lipid membrane. The forming tetramer led melittin to be active and caused the destruction of the lipid membrane by the toroidal pore. Melittin has an affinity for many types of biological bilayer phospholipid model systems. The disruption of the lipid membrane depends upon the geometry and thickness of the lipid bilayer, the membrane composition and the polar head group charge [14,27].

Melittin has demonstrated in vitro antiviral activities in certain diverse families of viruses such as *Arenaviridae*, *Flaviviridae*, *Herpesviridae*, *Orthomyxoviridae*, *Picornaviridae*, *Pneumoviridae*, *Rhabdoviridae* and *Virgaviridae*. A variety of possible antiviral mechanisms of the melittin peptide, such as impeding viral multiplication, decreasing the expression levels of viral mRNAs, inducing conformational changes in the viral genome, deactivation of the viral packaging, attenuation of the viral cytopathic effects and inhibition of viral-induced cell fusion, was demonstrated [19,28,29,30,31,32]. Among these antiviral mechanisms, the highlighted action of melittin was illustrated primarily through virolysis or virucidal activity [31].

In previous studies, bee venom and melittin were investigated for their antiviral activities. These compounds at non-toxic concentrations were found to significantly inhibit HSV–GFP after incubation of the compounds with a viral suspension at 4 °C for 30 min. The results showed that the ED_50_ values of bee venom and melittin against GFP-fused HSV were 1.52 ± 0.11 and 0.94 ± 0.07 µg/mL, respectively. Moreover, the GFP-fused HSV treated with melittin (2.0 µg/mL) was inoculated into Vero cells, and this experiment showed a marked reduction in GFP expression when compared to the untreated group at 24 h post infection. Notably, this effect appeared to have occurred due to the involvement of the direct interaction of melittin with the virus surfaces. Melittin appeared to have bound to the surface of the enveloped viruses, and these interactions caused the destabilization of the virus structure and the inactivation of the viral particles. Thus, the antiviral properties of bee venom and melittin were mainly explained to be a consequence of the virucidal mechanism [31]. Melittin also exhibited this action in a broad panel of viruses, including enveloped viruses such as influenza A virus (GFP-fused influenza A PR8), vesicular stomatitis virus (GFP-fused VSV) and respiratory syncytial virus (GFP-fused RSV). Additionally, bee venom and melittin peptide also inhibited non-enveloped viruses such as Enterovirus-71 (GFP-fused EV-71) and coxsackievirus (GFP-fused coxsackievirus H3) [31].

In addition, melittin is a cationic molecule that has a high affinity to neutral and anionic lipid membranes. Thus, for melittin, the act of being bound to lipid vesicles is fast and occurs rapidly, within milliseconds. Consequently, it forms aggregates or pores in the membrane [33]. These actions are the primary activities of melittin, and these properties are highly effective against a broad spectrum of pathogenic microorganisms, including bacteria and fungi. These actions are also known to have a killing effect on viruses, protozoa and cancer cells [17]. Moreover, melittin-related peptides, namely the frog-derived RV-23 and AR-23, exhibited strong antiviral activity against the sandfly fever Naples virus by blocking the virus–cell interaction through interaction with heparan sulfate [34]. Thus, melittin might block the binding of HSV particles with heparan sulfate receptor molecules.

MEL-AM and MEL-AF displayed a moderate degree of inhibition of HSVs when the peptides were added simultaneously as the HSVs attached to the cells. This result indicated that the interaction of melittin with the viral envelope was able to interfere with the binding or uptake of the viruses by the cells. The structure of the viral envelope is created from the cellular membrane, as the mature virions are released from the infected cell via budding. Many glycoproteins, such as gD, gB and the heterodimer gH/gL, are required and play an important role in the initial stage of infection and during the process of cell-to-cell spreading. These molecules are sufficient to facilitate HSV attachment to the cells with a receptor, which is followed by the fusion of the viral outer envelope with the plasma membrane of the infected cell [5,35]. Thus, the disruption or inactivation of the envelope lipid membrane or glycoproteins might be the main target of melittin in reducing the infection of HSVs. A number of reports have revealed that a polycationic peptide like melittin known as dermaseptin displayed a similar antiviral activity. Dermaseptin is secreted by the skin of amphibians and is composed of 28–34 amino acid residues that exhibit anti-HSV-2 activities. Dermaseptin and its derivatives displayed a moderate decrease in the infectivity of HSV-2 when the virus was treated during cell adsorption. However, the co-incubation of the cell-free virus and dermaseptin peptides before infection resulted in a strong reduction in viral infectivity [36]. In addition, the antiviral action of melittin-loaded liposomes and melittin-loaded immunoliposomes were studied, and the results revealed that the melittin-loaded particles were capable of inhibiting fish viral hemorrhagic septicemia rhabdovirus (VHSV) infectivity by 89.9% and 95.2%, respectively. This action of the melittin-loaded immunoliposomes was higher and illustrated via the direct inactivation of the virus [37].

Meanwhile, at the same time, the inhibition of both types of HSVs was less than 50% when the treatment was administered after HSV attachment to the cells. Notably, melittin displayed a low activity to inhibit the HSVs, as was shown using the plaque reduction assay, but a reduction in the cytopathic effects was also detected, especially in HSV-2; the plaque size diameter was reduced significantly with 5 µg/mL of MEL-AM and MEL-AF. Microscopic examination showed that the cytopathic effects of the HSVs, such as aggregation, nuclear enlargement and cell rounding, were reduced after treatment when compared with the untreated cells. Thus, melittin might interrupt the spread of viruses from infected cells to neighboring uninfected cells [38].

These results correlate with those of previous reports that have stated that melittin was shown to display inhibitory action against the Junín virus and HSVs. The yields of HSV-1 and HSV-2 were reduced by 80% when infected cells were treated with 3 µM of melittin for 24 h post infection (hpi). Furthermore, the Junín virus was found to be more sensitive to melittin, with an inhibitory action of 99% in the melittin-treated group. Nevertheless, non-toxic doses of melittin should be considered for further use since melittin was found to be quite toxic to Vero cells [18]. Additionally, a study of melittin and an analogue of melittin also inhibited cell fusion in Vero cells caused by HSV-1 mutant viruses including MP (*MP*), KOS (*sys20*) and KOS (*FFV*) strains that harbor the *syn1* mutation in glycoprotein K. Moreover, the presence of melittin at non-cytotoxic concentrations interfered with the activity of Na^+^, K^+^ ATPase in infected Vero cells and the distribution of trans-membrane ion gradients that impeded HSV-1-induced cell fusion [39,40]. These findings suggest that MEL-AM and MEL-AF might contain antiherpetic activity with a different mechanism from standard nucleoside drugs like acyclovir. Hence, these peptides might be suitable or have potential to inhibit and reduce the concerns that are associated with the development of drug-resistant viruses [41]. Although melittin has been reported to be able to inhibit various types of viruses, the degree of cytotoxicity of this peptide is the main concern with regard to its use in both laboratory and clinical trials. The clarification of the antiviral mechanisms of melittin in this study has provided evidence to support the use of melittin as a therapeutic agent for HSV infection.

In this study, the anti-inflammatory activities of melittin were assessed using LPS-stimulated murine macrophage cells. Bacterial lipopolysaccharide (LPS) is known to be able to stimulate the production of many proinflammatory mediators and proteins, such as iNOS, TNF-α, IL-6 and COX-2, in various inflammatory cells and to cause the physiological responses of inflammation, sepsis and stroke [42,43,44]. Based on this information, the anti-inflammatory activities of melittin might be explained by the inhibition of the nitric oxide production generated from arginine, mainly by inducible nitric oxide synthase (*iNOS*). In this study, MEL-AM and MEL-AF at non-cytotoxic concentrations could inhibit NO in a dose-dependent manner. Although NO plays an important role in the maintenance of the vascular and immune system, high amounts of oxidative NO obtained from the overexpression of *iNOS* in the inflammatory cells can cause pathophysiology in a variety of diseases and conditions, such as carcinogenesis and inflammation [45,46]. In addition, COX-2 and IL-6 are the most important pro-inflammatory mediators [47]. The extensive production of these mediators can be harmful to heathy organs.

The MEL-AM and MEL-AF melittin peptides demonstrated the suppression of the inflammatory genes *iNOS*, *COX-2* and *IL-6* after RAW 264.7 cells were stimulated with LPS and treated with melittin for 3 h in this study. It has been reported that melittin from *A. melifera* also reduced one of the intracellular signaling pathways, the mitogen-activated protein kinase (MAPK) pathway, and this pathway is associated with pro-inflammatory responses. The activation of the MAPK pathway was triggered by LPS and affected p38 MAPK, ERK2 and JNK in terms of both their mRNA and protein levels [21,44]. Therefore, the reduction in these pro-inflammatory mediators caused by MEL-AM and MEL-AF might supported the deactivation of the MAPK pathway in LPS-induced RAW 264.7 cells, which then would result in the inhibition of their downstream mRNA expression. A previous study also reported that whole bee venom has the ability to inhibit (NO) production in a model of lipopolysaccharide (LPS)- and sodium-nitroprusside-stimulated-RAW 264.7 macrophages [48]. Thus, the anti-inflammatory effect of bee venom was exhibited by melittin, as the main bioactive component present in bee venom. In addition, bee venom and melittin could suppress LPS-stimulated nitric oxide and inducible NO synthase (iNOS) expression in the pretreatment of BV2 microglia cells. Moreover, bee venom and melittin also suppressed the activation of nuclear factor kappa B (NF-κB) by blocking the degradation of IκBα and the phosphorylation of c-Jun N-terminal kinase (JNK) and Akt, which in turn affected the production of nitric oxide and proinflammatory cytokines such as interleukin (IL)-1β, IL-6 and tumor necrosis factor (TNF)-α [49]. Therefore, this study demonstrated that melittin contains both anti-HSV and anti-inflammatory activities and could be a substance with high potential for further development as an anti-HSV and anti-inflammation agent.

## 5. Conclusions

In summary, this study demonstrated that the MEL-AM and MEL-AF peptides effectively displayed antiviral activities against HSV-1 and HSV-2, mainly via the direct inactivation of the viral particles. The interaction between melittin and the viral particles resulted in an interference at the initial stage of the HSV infection when viral attachment to the cells took place. In addition, the MEL-AM and MEL-AF peptides represented a high potential to inhibit the nitric oxide production in LPS-stimulated RAW 264.7 macrophage cells. The melittin peptides were also found to down-regulate the expressions of *iNOS*, *COX-2* and *IL-6* mRNA. The highest inhibition of *IL-6* mRNA expression was found after treatment with MEL-AM and MEL-AF. Therefore, the MEL-AM and MEL-AF melittins have displayed clear potential to be developed as therapeutic agents to treat infectious HSV diseases and as anti-inflammatory agents in the future.

## Data Availability

The original contributions presented in the study are included in the article. Further inquiries can be directed to the corresponding author.
